# The utility of the integrated behavior change model as an extension of the theory of planned behavior

**DOI:** 10.3389/fpsyg.2022.940777

**Published:** 2022-08-17

**Authors:** Kimberly R. More, L. Alison Phillips

**Affiliations:** ^1^Department of Psychology, University of Dundee, Dundee, United Kingdom; ^2^Department of Psychology, Iowa State University, Ames, IA, United States

**Keywords:** theory of planned behavior, integrated behavior change model, physical activity, stage of change, dual process cognition

## Abstract

**Introduction:**

There are several widely used theories of health behavior change, which mostly utilize the social cognitive approach. These theories tend to posit that intention is a direct predictor of behavior, do not include automatic influences on behavior, and propose a one-size-fits-all theory for both initiators and maintainers. However, the intention-behavior gap is a well-observed phenomenon, researchers have highlighted that both automatic and reflective factors promote behavioral engagement, and predictors of behavior have been shown to differ between initiators and maintainers—three issues that necessitate theory advancement. To that end, the present research compares the utility of the Integrated Behavior Change Model (IBCM) – a social cognitive model that includes automatic factors involved in behavioral engagement and a moderator of the intention-behavior gap – to its theoretical predecessor, the Theory of Planned Behavior (TPB). Further, the relevance of the IBCM factors for predicting exercise behavior is compared in initiators versus maintainers.

**Method:**

Participants were 494 US undergraduates. Participants reported on variables from the IBCM (and TPB) at baseline and reported on their exercise behavior in two surveys at seven- and 14-days post-baseline.

**Results:**

Findings supported the first hypothesis that the IBCM would be more relevant for initiators in comparison with maintainers, using structural equation modeling. Specifically, only the paths between intrinsic motivation and affective attitude, affective attitude and intention, and intention and behavior were reliably found for maintainers. For initiators, the aforementioned paths were also reliably supported and the additional following paths were also supported: intrinsic motivation and perceived behavioral control, perceived behavioral control and intention, and intention and action planning. However, results did not support the second hypothesis that the IBCM would predict significantly more variance in behavior than its theoretical predecessor, the TPB. Specifically, the addition of action planning, implicit attitude, implicit motivation, and the interaction between intention and action planning only predicted an additional 0.3% (*p* < 0:05) of the variance in exercise behavior above and beyond intention.

**Conclusion:**

Results highlight the continued need for theoretical refinement in terms of delineating mechanisms of initiation and maintenance and the need for further development in terms of improving upon current predictions of behavior engagement and change.

## Introduction

The World Health Organization reports that worldwide 1.4 billion adults do not engage in sufficient levels of physical activity (WHO, 2020). This is problematic given that sufficient levels of physical activity engagement can decrease the risk of all-cause mortality as well as the onset of certain chronic illnesses such as hypertension, type 2 diabetes, and some site-specific cancers (e.g., colon). The fields of health psychology and behavioral medicine utilize many theoretical models to understand health behavior and health behavior change (Smedslund, 2000). Most of these theories take the social cognitive approach, which hypothesizes that intention is a direct proximal predictor of behavioral action, neglect to include the influence of automatic constructs on behavior, and generally do not differentiate between mechanisms of initiation and maintenance – specifying an invariant theory of behavior. However, the intention-behavior gap is a well-observed phenomenon (Sheeran and Webb, 2016), both automatic and reflective constructs have been shown to influence behavioral engagement (Rebar et al., 2016), and predictors of behavior have been shown to differ between initiators and maintainers (Phillips et al., 2016) – three issues that require theory advancement to promote more well-rounded, theoretically-based interventions. Using an exploratory approach, the focus of the present research is to compare, conceptually and statistically, two theories of health behavior change – for exercise initiators and maintainers – which are rooted in the link between intentions and behavior: The Integrated Behavior Change Model (IBCM; Hagger and Chatzisarantis, 2014b) and its theoretical predecessor, the Theory of Planned Behavior (TPB; Fishbein and Ajzen, 2011). Moreover, this research will compare the utility of the IBCM for predicting behavior against the TPB for initiators.

### The theory of planned behavior

**FIGURE 1:**
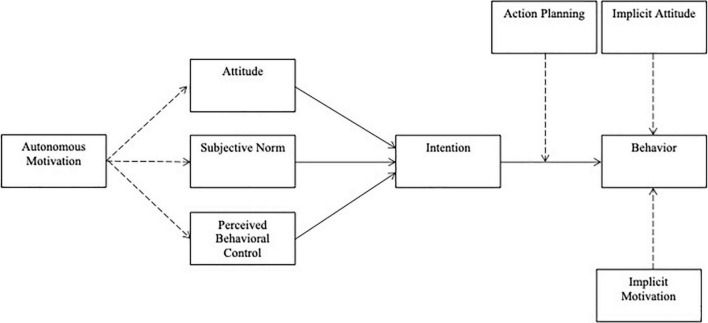
The theory of planned behavior and the integrated behavior change model. The Theory of Planned Behavior is represented by the solid lines only. The Integrated Behavior Change model is an extension of the Theory of Planned Behavior and encompasses the entirety of the figure.

The TPB (Fishbein and Ajzen, 2011) specifies that behavior is proximally predicted by intentions and that intention is proximately predicted by attitudes, subjective norms, and perceived behavioral control (Table 1 and Figure 1). Within the TPB, all constructs are reflective in nature. Moreover, the TPB includes three mediational hypotheses whereby attitudes, subjective norms, and perceived behavioral control predict behavior through intentions (Table 1). In general, *attitudes* refer to an individual’s beliefs about whether a behavior is favorable or unfavorable (Ajzen, 1991). Attitudes can be delineated into affective and instrumental types – a distinction that is not explicitly made in the TPB. *Affective attitudes* are emotion-based, whereas *instrumental attitudes* are based on thoughts about the costs and benefits associated with a behavior (Hamilton and Johnson, 2020). Generally, *subjective norms* refer to an individual’s perception of the social pressure surrounding behavioral performance (Ajzen, 1991). Subjective norms can be further delineated into descriptive and instrumental norms – similar to attitude types this distinction is not explicitly made by the TPB. *Descriptive norms* are an individual’s perception of the behavior of others, whereas *injunctive norms* are an individual’s perception of perceived pressure to engage in a behavior (Okun et al., 2002). *Perceived behavioral control* refers to an individual’s assessment of how easy or difficult it would be to engage in a behavior (Ajzen, 1991). Finally, *intentions* reflect an individual’s motivation to engage in a behavior (Ajzen, 1991). Intentions can vary in quality with higher quality intentions (e.g., intentions that are based on goals that are promotion versus prevention focused, autonomy versus control focused, and mastery versus performance focused) being more likely to lead to behavioral performance (Sheeran and Webb, 2016).

**TABLE 1:** Theory of Planned Behavior predictions for positive health behaviors.

		Independent variable	Dependent variable	Mediator	Prediction
**Direct effects**					
	1	Attitude	Intention	–	Effect (+)
	2	Subjective norm	Intention	–	Effect (+)
	3	Perceived behavioral control	Intention	–	Effect (+)
	4	Intention	Behavior	–	Effect (+)
**Mediated effects**					
	1	Attitude	Behavior	Intention	Effect (+)
	2	Subjective norm	Behavior	Intention	Effect (+)
	3	Perceived behavioral control	Behavior	Intention	Effect (+)

The TPB (Fishbein and Ajzen, 2011) has been criticized in part for not addressing the robustly observed intention-behavior gap (e.g., Sniehotta et al., 2014). Specifically, approximately 50% of intentions do not get translated into behavioral action (Sheeran and Webb, 2016). Perhaps unsurprisingly, meta-analyses of experimental studies have found that a medium-to-large change in manipulated intentions only leads to a small-to-medium change in behavior (Webb and Sheeran, 2006; Rhodes and Dickau, 2012). The intention-behavior gap is largely due to *inclined abstainers*, those who intend to change their behavior but fail to do so (Godin and Conner, 2008). Not acting on intentions can be due to barriers such as forgetting intentions, failing to engage in preparatory behaviors, or missing behavioral opportunities (Sheeran and Webb, 2016). Considering this, forming if-then plans (i.e., action planning/implementation intentions) has been suggested as an effective means of reducing the intention-behavior gap. Specifically, a meta-analysis (Gollwitzer and Sheeran, 2006) found that action planning is strongly related to actual behavior (*d* = 0.65), and that this effect did not significantly differ based on study design (observational: *d* = 0.70; experimental: *d* = 0.65) or on the type of outcome measure (self-report: *d* = 0.63; objective: *d* = 0.67). Specifically, action plan formation predicted greater detection (*d* = 0.72) and attention (*d* = 0.72) to specified cues as well as behavioral opportunities for action.

### The integrated behavior change model

The IBCM (Hagger and Chatzisarantis, 2014b) expands upon the TPB by including action planning as a moderator of the intention-behavior gap, by including automatic processes alongside reflective processes, and by introducing a humanistic approach with the inclusion of autonomous motivation derived from Self-Determination Theory. Specifically, the IBCM adds (1) autonomous motivation as a predictor of attitudes, subjective norms, and perceived behavioral control, (2) implicit attitudes and implicit motivation (automatic factors) as direct predictors of behavior, and (3) action planning as a moderator of the intention-behavior gap (Figure 1; Hagger and Chatzisarantis, 2014b). Preliminary support for this theory has been given with regard to both simple behaviors such as sunscreen use as well as more complex behaviors such as fruit and vegetable consumption (Hagger et al., 2017; Hamilton et al., 2017; Brown et al., 2018; Caudwell et al., 2019; Shannon et al., 2019). To date, the full IBCM has yet to be tested with physical activity or exercise as an outcome (although this extension was theoretically proposed in 2014 by Hagger and Chatzisarantis, 2014a). Preliminary observational studies using the IBCM have *not* supported the moderating effect of action planning on the relationship between intention and behavior. This is the case for research examining sugar consumption, sun safety behaviors, and fruit and vegetable consumption (Hagger et al., 2017; Hamilton et al., 2017; Brown et al., 2018). That is, the intention-behavior gap has not been reduced by the inclusion of action planning in observational research using the IBCM as a guiding theory.

Preliminary findings have supported the direct relationships between autonomous motivation and attitudes, subjective norms, and perceived behavioral control regarding sugar consumption, fruit and vegetable consumption, pre-drinking behavior, sun safety behavior, and self-management of mental health (Hagger et al., 2017; Hamilton et al., 2017; Brown et al., 2018; Caudwell et al., 2019; Shannon et al., 2019). The mediated relationship between autonomous motivation and intentions through attitudes, subjective norms, and perceived behavioral control were also supported for sugar consumption, fruit and vegetable consumption, and sun safety behaviors (Hagger et al., 2017; Hamilton et al., 2017; Brown et al., 2018; Shannon et al., 2019). However, the relationship between autonomous motivation and pre-drinking intention was only mediated by attitudes (Caudwell et al., 2019). In addition, there were no significant indirect effects regarding self-management of mental health (Shannon et al., 2019).

Finally, findings on the utility of the addition of implicit attitudes and implicit motivation as direct predictors of behavior within the IBCM are sparse. First, a literature search revealed only one empirical test of implicit attitudes within the framework, which supports the direct relationship between implicit attitudes about sugar and actual sugar consumption (Hagger et al., 2017). Second, implicit motivation as a direct predictor of behavior – as suggested by Hagger and Chatzisarantis (2014b) – has not been included in any test of the IBCM to date.

### Stage of change

The absence of a significant moderating effect of action planning in tests of the IBCM does not negate the important role of action planning in intention fulfillment with regard to both the observational and experimental literature (Gollwitzer and Sheeran, 2006). The mechanisms of behavioral engagement have been theorized to differ between individuals who are just starting a behavior (i.e., initiators) versus those who have been engaging in a behavior for some time (i.e., maintainers; Rothman, 2000; Rothman et al., 2009; Rhodes et al., 2021). Specifically, behavioral intention is thought to be a mechanism of initiation, and action planning is a means of action control to translate behavioral intention into behavior, which may be less relevant for maintainers (Rhodes et al., 2021). Previous tests of the IBCM for health outcomes do not recruit participants based on their stage of behavior change (Hagger et al., 2017; Hamilton et al., 2017; Brown et al., 2018; Caudwell et al., 2019; Shannon et al., 2019). Including participants from both the initiation and maintenance phase is a limitation and likely skews the direct linear effect of intentions on behavior as well as the moderating effect of action planning due to the shift to automatization of behavior when an individual has repeated behavioral experiences (Sheeran and Webb, 2016; Sheeran et al., 2017). Indeed, intentions have been shown to predict exercise behavior in initiators but not maintainers (Phillips et al., 2016). Additionally, other social cognitive models of health engagement have started to delineate the mechanisms of behavior for initiators versus maintainers (e.g., Health Action Process Approach, Schwarzer and Luszczynska, 2008; the Commonsense Model of Self-Regulation, Phillips et al., 2013).

### Purpose of the present research

The purpose of the present research is twofold. First, the utility of the predictions made by the IBCM (Figure 1) – including automatic factors and moderation of the relationship between intention and behavior by action planning – will be compared between initiators and maintainers of physical activity. It was predicted that this model would fit better for initiators in comparison with maintainers. Second, prediction of physical activity behavior by the IBCM will be compared to prediction by its theoretical predecessor, the TPB, for initiators. This hypothesis was tested for initiators only as neither the TPB, nor the IBCM, includes constructs that have been proposed to be critical for maintenance (Rhodes et al., 2021).

## Materials and methods

### Participants and procedures

Participants were recruited from a Midwestern university in the United States and were eligible to participate if they were 18 years of age or older and participated in physical activity, at least sometimes. Non-exercisers were not eligible to participate in the present study, because they were not in the initiation or maintenance phase of behavior change. In addition to this, National Collegiate Athletic Association (NCAA) athletes were ineligible for participation because they have at least some of their exercise sessions scheduled by an external source. Participants reported their age, exercise stage of change, and NCAA status at pre-screen. A total of 21 participants were excluded at the pre-screen for not meeting the eligibility criteria. Eligible participants were directed to baseline and were asked to complete two weekly assessments of their exercise behavior at seven- and 14-days post-baseline. Participants were compensated with course credit. All procedures were approved by the institutional review board prior to data collection, and informed consent was collected from all participants via a checkbox in the online baseline survey. Hypotheses and analyses were pre-registered on the Open Science Framework (https://osf.io/78fuh), study termination because of the announcement of the COVID-19 pandemic was also registered on the Open Science Framework (https://osf.io/4b7gf). It should be noted that the analysis type for the second hypothesis was updated after data collection to reflect a more appropriate methodology. Thus, the analysis of the second hypothesis should be considered exploratory in nature.

### Measures

#### Pre-screen

Participants reported their age, gender, race, ethnicity, and NCAA membership. Additionally, participants reported their *stage of change* regarding exercise (Prochaska and Velicer, 1997). Participants were asked to ‘Please tell us which option most closely fits you currently’ (Note: ‘Regular exercise’ = 3 or more times per week for at least 30 min at moderate or greater intensity each time). Response options were: (1) ‘I currently do not exercise and I do not intend to start’, (2) ‘I currently do not exercise, but I am thinking about starting’, (3) ‘I currently exercise some, but not regularly (regularly is 3x per week or more)’, (4) ‘I currently exercise regularly, but have only begun doing so within the past 6 months’, and (5) ‘I currently exercise regularly, and I have been doing so for longer than 6 months’. These options correspond to pre-contemplation, contemplation, preparation, action, and maintenance, respectively. Participants who were in the pre-contemplation or contemplation stages were not eligible for participation because they did not engage in exercise. Participants in the preparation or action stages of behavior change were classified as ‘initiators’, and participants in the maintenance phase were classified as ‘maintainers’.

#### Baseline

*Autonomous motivation* was assessed using the Behavioral Regulation in Exercise Questionnaire – 3 (Markland and Tobin, 2004; Wilson et al., 2007). Three types of autonomous motivation were measured with four items each: (1) integrated (e.g., ‘I exercise because it is consistent with my life goals’), (2) identified (e.g., ‘It’s important to me to exercise regularly’), and (3) intrinsic (e.g., ‘I enjoy my exercise sessions’).

*Explicit attitudes* were measured in terms of both instrumental and affective attitudes (Rhodes and Courneya, 2010). All items were preceded by the following stem: ‘Over the next 2 weeks, engaging in physical activity on a regular basis would be…’. Affective attitudes were assessed using three items ranging from: (1) ‘boring’ to ‘interesting’, (2) ‘unenjoyable’ to ‘enjoyable’, and (3) ‘stressful’ to ‘relaxing’. Instrumental attitudes were assessed using three items ranging from: (1) ‘harmful’ to ‘beneficial’, (2) ‘useless’ to ‘useful’, and (3) ‘foolish’ to ‘wise’.

*Subjective norms* were measured in terms of both injunctive and descriptive norms (Rhodes and Courneya, 2010). Injunctive norms were measured using the following two items: (1) ‘Most people in my social network want me to exercise regularly in the next 2 weeks’, and (2) ‘Most people in my social network would approve if I exercised regularly in the next 2 weeks’. Descriptive norms were measured using the following three items: (1) ‘Most of my friends exercise regularly’, (2) ‘Most of my family members exercise regularly’, and (3) ‘Most of my college peers exercise regularly’. The third item was adapted from the original measure, which specified co-workers in lieu of college peers.

*Perceived behavioral control* was assessed using three items from Rhodes and Courneya (2010): (1) ‘How confident are you that you will be able to exercise regularly in the next 2 weeks’, (2) ‘How confident are you over the next 2 weeks that you could overcome obstacles that prevent you from exercising regularly’, and (3) ‘I believe that I have the ability to regularly exercise in the next 2 weeks’.

*Intention* was measured using one item from Rhodes and Courneya (2010): ‘Over the next 2 weeks, I intend to exercise _________times per week’.

*Action planning* was measured using four items from Sniehotta et al. (2005). Four items followed the stem: ‘I have made a detailed plan regarding…’. Items were: (1) ‘when to exercise’, (2) ‘where to exercise’, (3) ‘how to exercise’, and (4) ‘how often to exercise’.

*Implicit attitudes and motivation* were assessed using two Implicit Association Tests (IATs) created in the iatgen program (Carpenter et al., 2019). For the attitude IAT, stimuli from the categories ‘good’ (i.e., pleasure, enjoy, happy), ‘bad’ (i.e., pain, horrible, sadness), ‘exercise’ (i.e., active, fitness, workout), and ‘sedentary’ (i.e., inactive, seated, sitting) were used. Terminology used for the exercise and sedentary stimuli were adapted from Banting et al. (2009) to reflect neutral sedentary words – in lieu of words with negative connotations. This was done as even the most active individuals engage in sedentary behaviors, such as sitting down in lieu of standing, at least some of the time and over-engagement in sedentary behaviors poses risk even to individuals who exercise (e.g., Keadle et al., 2014). Thus, words like ‘lazy’ and ‘sluggish’ are likely not reflective of all sedentary behavior engagement. For the motivation IAT, stimuli were adapted from Keatley et al. (2014): self (i.e., me, myself), not self (i.e., it, that), autonomous motivation (i.e., choice, free, spontaneous, willing, authentic), and controlled motivation (i.e., pressured, restricted, forced, should, controlled). IAT order was randomized using a random number generator. Additionally, within IAT, left or right starting position was also randomized within each IAT. This is standard practice and implemented automatically by the iatgen program (Carpenter et al., 2019).

Two *random response checks* were administered at baseline to detect careless responding as this response pattern has been shown to drastically alter effect sizes (Credé, 2010). Participants who failed either of these checks were excluded from all analyses (*n* = 17).

#### Weekly surveys

*Physical activity behavior* was measured at baseline and at each of the two weekly surveys using the International Physical Activity Questionnaire (Booth, 2000). Participants self-reported the number of minutes that they engaged in moderate and vigorous physical activity over the previous seven days. A composite score of moderate and vigorous physical activity was created for each timepoint. Weekly assessments were utilized if they were completed within 48 hours of administration and were emailed to participants at nine o’clock in the morning, with a follow-up email being administered 24 hours prior to the 48-hour deadline.

### Statistical analyses

Power analysis for the proposed paths within the IBCM was conducted *a priori* using Monte Carlo simulations in Mplus, using α = 0.05 and 1,000 bootstrapped samples (Muthén and Muthén, 2012).

Participants’ implicit attitudes and motivation IATs were scored using the Greenwald et al. (2003) scoring algorithm through the iatgen shiny app program (Carpenter et al., 2019). This scoring resulted in a D-score, with higher scores indicating more positive implicit attitudes and more autonomous motivation and lower scores indicating less positive implicit attitudes and more controlled motivation.

Data were examined for multivariate outliers on all models, separately for initiators and maintainers using Mahalanobis distances (*p* < 0.001). Multivariate outliers were removed and the analysis reconducted until there were no outliers remaining. A multiverse approach (Steegen et al., 2016) was taken where the baseline data, week one data, and week two data were all analyzed with and without multivariate outliers. Main results are reported with the inclusion of multivariate outliers and baseline data. Deviations in results according to the multiverse approach are also reported.

Self-Determination Theory hypothesizes that motivation can vary in terms of autonomy. Therefore, a maximum likelihood exploratory factor analysis with an oblique rotation was used to determine whether all types of autonomous orientation (i.e., intrinsic, identified, and integrated) should be combined into one scale. Parallel analysis was used to determine the appropriate number of factors for extraction (Zwick and Velicer, 1986).

The first analysis, which compares the utility of the IBCM between initiators and maintainers, was conducted using multi-group recursive structural equation modeling in Mplus to test the relationships between both observed and latent variables (Muthén and Muthén, 2012). The second analysis, which compares the IBCM to its theoretical predecessor, the TPB, was analyzed using hierarchical linear regression to determine whether implicit motivation, implicit attitudes, action planning, and the interaction between action planning and intention add to the prediction of behavior above and beyond intention. This contrasts with the pre-registered analysis, which specified comparing the two theories using structural equation modeling. This pre-registered plan was deviated from because Mplus does not allow for comparing models with different observed variables (Mplus Discussion, 2008).

## Results

### Preliminary results

For the power analysis, as suggested by Muthén and Muthén (2002), path parameter estimates were obtained from a previous meta-analysis conducted by Hagger and Chatzisarantis (2014a). However, implicit attitudes, implicit motivation, and action planning were not assessed in the meta-analytic study, and therefore *β* (standardized beta coefficients) values for the relationship between these variables and behavior were estimated using a sensitivity analysis from previous tests of the IBCM in terms of both weakest and strongest reported values (i.e., Hagger et al., 2017; Hamilton et al., 2017; Brown et al., 2018; Caudwell et al., 2019; Shannon et al., 2019). Parameter estimates were expressed as (*β*) to account for shared variance between a set of predictors with a given outcome variable with residual variance being calculated using the formula: 1 – Σ(*β*^2^). The primary power analysis suggested that 300 initiators and 300 maintainers were needed to be sufficiently powered across paths, however, due to the COVID-19 pandemic, data collection was terminated early as lockdown resulted in many participants leaving the area and having their routines disrupted. Online data collection after this point was deemed not feasible as the measured variables and the relationships between them would almost certainly have been affected by the pandemic. An updated power analysis for the IBCM for 287 initiators and 207 maintainers was conducted. For initiators, the observed power was as follows: (1) autonomous motivation and attitude, 100%, (2) autonomous motivation and subjective norms, 65.1%, (3) autonomous motivation and perceived behavioral control, 100%, (4) attitude and intention, 100%, (5) subjective norm and intention, 22.3%, (6) perceived behavioral control and intention, 98.9%, (7) intention and behavior, 66.1–96.9%, (8) implicit attitudes and behavior 47.7–97.1%, (9) implicit motivation and behavior, 46.5–96.2%, (10) action planning and behavior, 12.3–99.6%, and (11) action planning*intention and behavior, 14.5–67.9%. For maintainers, the observed power was: (1) autonomous motivation and attitude, 100%, (2) autonomous motivation and subjective norms, 52.8%, (3) autonomous motivation and perceived behavioral control 99.9%, (4) attitude and intention, 100%, (5) subjective norms and intention, 18.4%, (6) perceived behavioral control and intention, 96.1%, (7) intention and behavior, 52.2–93.8%, (8) implicit attitudes and behavior, 36.9–89.3%, (9) implicit motivation and behavior, 35.4 and 89.1%, (10) action planning and behavior, 10.9–97.4%, and (11) action planning*intention and behavior, 10.3–53.7%.

A total of 17 participants were excluded from data analysis because they failed at least one of two random response checks at baseline leaving a total of 494 participants (i.e., 287 initiators and 207 maintainers). Participants were 19.31 years of age on average (*SD* = 1.77), and most participants self-identified as female using she/her pronouns (54.7%). Most participants also self-identified as Caucasian (86%). For the weekly data, 339 participants completed the first survey and 323 completed the second survey. Maintainers engaged in more moderate and vigorous physical activity in comparison to initiators, at baseline [*t*(473) = −3.70, *p* < 0.001], week one [*t*(353) = −4.08, *p* < 0.001], and week two [*t*(336) = −3.43, *p* = 0.001]. Additionally, maintainers (*M*_*months*_ = 28.79, *SE* = 0.58) had been engaging in regular exercise significantly longer than initiators (*M*_*months*_ = 5.67, *SE* = 2.05; [*t*(474) = −12.67, *p* = < 0.001]).

Missing data was relatively sparse as the survey reminded (but did not force) participants to respond to unanswered questions. For predictor variables, there was only one missing item for injunctive subjective norms. For the outcome variable of exercise there were 19 cases of missing data at baseline, eight cases for week one, one case for week two. Missing data represented at least 50% of each scale. Therefore, multiple imputation was not conducted as the proportion of missing data was too large to impute (Garson, 2015).

A maximum likelihood exploratory factor analysis with an oblique rotation – allowing for factors to be correlated – was conducted to determine whether all autonomous motivation types should be combined into one factor. A parallel analysis (Zwick and Velicer, 1986) revealed that it was appropriate to extract two factors. Both factors also met the eigenvalue greater than one threshold (i.e., Factor 1 = 6.09; Factor 2 = 1.28). The first factor accounted for 50.73% of the total variance and was made up of the items from the integrated and identified subscales of the BREQ-3 (Table 2). The second factor explained 10.69% of the total variance and was made up of the items from the intrinsic motivation scale. Due to intrinsic motivation being the most prototypical form of autonomous motivation, and the fact that it loaded separately from both identified and integrated motivation types, it was used as the measure for motivation in hypothesis 1.

**TABLE 2:** Exploratory Factor Analysis (EFA) pattern matrix.

Item	Factor 1 loading	Factor 2 loading
Integrated: I consider exercise a fundamental part of who I am	1.04	
Integrated: I consider exercise part of my identity	0.991	
Integrated: I consider exercise consistent with my values	0.623	
Identified: It’s important for me to exercise regularly	0.516	
Integrated: I exercise because it is consistent with my life goals	0.499	
Identified: I get restless if I don’t exercise regularly	0.436	
Intrinsic: I enjoy my exercise sessions		0.906
Intrinsic: I find exercising a pleasurable activity		0.876
Intrinsic: I exercise because it’s fun		0.717
Intrinsic: I get pleasure and satisfaction from participating in exercise		0.632

Items with cross loadings ≤ 0.2 were not included in the model.

For hypothesis 1, multivariate outliers were removed separately for initiators and maintainers as these groups were analyzed separately and have been shown to differ on mechanisms of behavior in past research (Phillips et al., 2016). For the relationship between the predictor intrinsic motivation and the outcomes of perceived behavioral control, explicit attitudes, and descriptive subjective norms, there was one multivariate outlier for initiators and no multivariate outliers for maintainers exceeding the critical value of 18.47 (*p* < 0.001). For the relationship of intentions being predicted by perceived behavioral control, explicit attitudes, and descriptive subjective norms, there were six multivariate outliers for both initiators and maintainers exceeding the critical value of 18.47 (*p* < 0.001). For the relationship between intention and action planning, there were three multivariate outliers for initiators and no multivariate outliers for maintainers exceeding the critical value of 13.82 (*p* < 0.001). Finally, for the prediction of exercise behavior from intention, action planning, implicit attitudes, and implicit motivation, the multivariate analysis was as follows (critical value: 20.52, *p* < 0.001): (1) with baseline exercise as the outcome, there were eight initiators and two maintainers with outlying values, (2) with week one exercise as the outcome, there were three initiators and four maintainers with outlying values, and (3) with week two exercise as the outcome, there were three initiators and four maintainers with outlying values.

For hypothesis 2, multivariate outliers were assessed at the critical value of 20.52 (*p* < 0.001) for behavior, intention, action planning, implicit attitude, and implicit motivation. Unlike hypothesis 1, this was done without the removal of outliers for preceding relationships in the IBCM. Multivariate outliers were as follows: baseline had 13 outliers, week one had seven outliers, and week two had six outliers.

### Hypothesis 1

#### Measurement model

The first hypothesis, that the IBCM would more accurately reflect the antecedents of physical activity for initiators in comparison with maintainers was first assessed by examining the measurement model (i.e., CFA) and recursive structural equation modeling in initiators only. The measurement model was conducted between the latent variables and their indicators (i.e., all variables except for intention, implicit attitudes and motivation, and behavior; Kline, 2005; Hoyle, 2012). In this model, the latent factor of attitudes was composed of both affective and instrumental attitudes and the latent factor of subjective norms consisted of both injunctive and descriptive norm indicators (Rhodes and Courneya, 2010). This model was not a good fit for the data [RMSEA = 0.09, CFI = 0.83, TLI = 0.80, χ^2^ (199) = 615.18, *p* < 0.001]. Although all factor indices significantly loaded onto their latent factor (*p* < 0.001), instrumental attitudes represented points of ill fit on the latent factor of attitudes (Hoyle, 2012). Specifically, all *R*^2^ values were equal to or lower than 0.32 (i.e., 0.21 – 0.32). Additionally, injunctive norms represented points of ill fit with the latent factor of subjective norms with all *R*^2^ values being equal to or below 0.20 (i.e., 0.09 – 0.20). Both instrumental attitudes and injunctive norm items explained less variance in their latent variables than either affective attitudes (*R*^2^ = 0.34 – 0.62) or descriptive norms (*R*^2^ = 0.08 – 0.84). Additionally, instrumental attitudes (*R*^2^ = 0.68 – 0.79) and injunctive norms (*R*^2^ = 0.80 – 0.91) had higher levels of residual item variance compared to affective attitudes (*R*^2^ = 0.28 – 0.66) and descriptive norms (*R*^2^ = 0.17 – 0.93), respectively. Thus, a second measurement model without the inclusion of instrumental attitude and injunctive norm items was conducted and fit the data well [RMSEA = 0.04, CFI = 0.99, TLI = 0.96, χ^2^(109) = 169.49, *p* < 0.001]. It should be noted that within this model the first descriptive norm item was a poor indicator of the latent construct of subjective norms (factor loading: 0.27, residual variance: 0.93, *R*^2^ = 0.07). However, this item was retained as it is part of a validated scale and the removal of the item did not improve the overall model fit [RMSEA = 0.05, CFI = 0.97, TLI = 0.96, χ^2^(95) = 156.78, *p* < 0.001].

A structural model was computed to determine whether action planning significantly moderated the intention behavior gap, as the analysis type in Mplus that allows for specification of interactions between latent and observed variables does not provide indices of model fit, which are necessary to compare models (Muthén, 2009). Results indicated that action planning did not moderate the intention behavior gap (*p* = 0.28). Considering this, the interaction term was removed from the model and hypothesis 1 was conducted using the type ‘general’, which allows for indices of model fit. Thus, action planning was instead specified as a variable linking intention and behavior, which has been done in previous structural equation modeling assessments of the IBCM (e.g., Hagger et al., 2017).

#### Structural equation model in initiators

Prior to comparing the IBCM between initiators and maintainers, the utility of this model in initiators only was established. Data is presented with the inclusion of multivariate outliers and with baseline physical activity data (RMSEA = 0.04, CFI = 0.95, TLI = 0.94).

Intrinsic motivation predicted perceived behavioral control (β = 0.26, *SE* = 0.07, *p* < 0.001) and affective attitude (β = 0.72, *SE* = 0.04, *p* < 0.001), but did not predict descriptive subjective norms (β = 0.11, *SE* = 0.06, *p* = 0.093). Second, perceived behavioral control (β = 0.18, *SE* = 0.06, *p* = 0.004), affective attitude (β = 0.24, *SE* = 0.06, *p* < 0.001), and descriptive subjective norms (β = 0.17, *SE* = 0.06, *p* = 0.003) all predicted intention to engage in physical activity, with affective attitudes being the strongest predictor. Intention predicted both action planning (β = 0.32, *SE* = 0.06, *p* < 0.001) and behavior (β = 0.15, *SE* = 0.06, *p* = 0.019). Action planning (β = 0.11, *SE* = 0.08, *p* = 0.115), implicit attitudes (β = 0.04, *SE* = 0.07, *p* = 0.549), and implicit motivation (β = −0.07, *SE* = 0.07, *p* = 0.289) did not predict behavior. All significant findings were consistent with the hypothesized direction (Table 3).

**TABLE 3:** Integrated Behavior Change Model predictions for positive health behaviors.

		Independent variable	Dependent variable	Mediator	Moderator	Prediction
**Direct effects**						
	1	Autonomous motivation	Attitude			Effect (+)
	2	Autonomous motivation	Subjective norm			Effect (+)
	3	Autonomous motivation	Perceived behavioral control			Effect (+)
	4	Attitude	Intention			Effect (+)
	5	Subjective norm	Intention			Effect (+)
	6	Perceived behavioral control	Intention			Effect (+)
	7	Intention	Behavior			Effect (+)
	8	Implicit attitude	Behavior			Effect (+)
	9	Implicit motivation	Behavior			Effect (+)
**Mediated effects**						
	1	Autonomous motivation	Intention	Attitude		Effect (+)
	2	Autonomous motivation	Intention	Subjective norm		Effect (+)
	3	Autonomous motivation	Intention	Perceived behavioral control		Effect (+)
	4	Attitude	Behavior	Intention		Effect (+)
	5	Subjective norm	Behavior	Intention		Effect (+)
	6	Perceived behavioral control	Behavior	Intention		Effect (+)
**Moderator effects**						
	1	Intention	Behavior		Action planning	Effect (+)

The multiverse analyses revealed several deviations from the main results. First, the relationship between intentions and behavior was non-significant, concerning week one exercise, when outliers were included. When outliers were excluded, the relationship between intention and behavior was significant in all models, except for the week 2 data, in which the data was not interpretable due to poor model fit. Thus, the relationship between intention and behavior was significant in four of the five interpretable models. Additionally, in the data with the removal of multivariate outliers, the relationship between descriptive subjective norms and intentions was non-significant in all interpretable models (i.e., except for week 2, which had poor model fit). Thus, the relationship between descriptive subjective norms and intention was significant in three of the five interpretable models. No other paths varied across the multiverse analysis in terms of either significance or directionality of the effect.

#### Comparison between initiators and maintainers

Hypothesis 1, that the IBCM would more accurately reflect the antecedents of behavior for initiators in comparison with maintainers, was first assessed by comparing the equivalence of the measurement model with fixed factor loadings with the measurement model with free factor loadings to assess metric invariance, which is a pre-requisite of multi-group structural equation modeling. The measurement model with fixed factor loadings for initiators and maintainers fit the data well [RMSEA = 0.05, CFI = 0.94, TLI = 0.94, χ^2^(242) = 417.77, *p* < 0.001]. The model that allowed free factor loadings for initiators and maintainers fit the data similarly well [RMSEA = 0.05, CFI = 0.95, TLI = 0.95, χ^2^(230) = 3328.17, *p* < 0.001]. The criteria of metric invariance were satisfied as the RMSEA values not differ substantially between models (Cheng and Rensvold, 2002). Specifically, The RMSEA values fit within each other’s confidence intervals (Fixed: 95% CI [0.045,0.063], Free: 95% CI [0.042,0.061]).

The model fit was assessed by comparing the model where factor loadings were fixed between initiators and maintainers, but paths were able to vary (i.e., unconstrained model), and a model where both factor loadings and path and mean parameters were fixed (i.e., constrained model). Across the six multiverse iterations, the unconstrained model fit better than the constrained model in five of the iterations. The unconstrained model was not supported in terms of baseline exercise when multivariate outliers were included. The overarching robustness of these results supports the notion that model paths and mean parameters are significantly different between initiators and maintainers.

**FIGURE 2:**
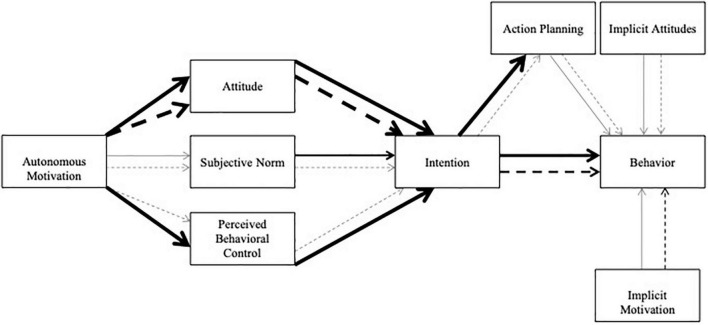
Supported paths across multiverse analysis for the Integrated Behavior Change Model weighted by level of support. Initiators are represented by the solid lines only. Maintainers are represented by dashed lines only. Thicker lines indicate support across more multiverse iterations than thinner lines.

Overall, across the five iterations of the multiverse analysis results were as follows (Figure 2): (1) intrinsic motivation predicted perceived behavioral control in five models for initiators and none of the models for maintainers, (2) intrinsic motivation predicted affective attitudes in five models for initiators and five models for maintainers, (3) intrinsic motivation predicted descriptive subjective norms in none of the models for initiators or for maintainers, (4) perceived behavioral control predicted intention in five models for initiators and none of the models for maintainers, (5) affective attitudes predicted intention in five models for initiators and five models for maintainers, (6) descriptive subjective norms predicted intention in two models for initiators and none of the models for maintainers, (7) intention predicted action planning in five models for initiators and none of the models for maintainers, (8) action planning predicted behavior in none of the models for initiators or for maintainers, (9) intention predicted behavior in four models for initiators and three models for maintainers, (10) implicit motivation predicted behavior in none of the models for initiators or for maintainers, and (11) implicit attitudes predicted behavior in none of the models for initiators and one model for maintainers. All significant paths were in the expected direction for initiators and maintainers (Table 3), except for the significant relationship between implicit attitude and behavior for maintainers in which worse attitudes resulted in more behavioral engagement with the week two data with the inclusion of multivariate outliers. Overall, these results further highlight how the IBCM is supported more for initiators in comparison with maintainers. However, there were no unique proximal predictors of behavior for initiators in comparison with maintainers overall.

### Hypothesis 2

The second hypothesis, that the IBCM would add unique direct predictors of behavior above and beyond the TPB, was assessed using linear regression. In all models, moderate and vigorous exercise behavior was the outcome (i.e., baseline, week one, or week two). Intention was entered into the first block, action planning, implicit attitude, and implicit motivation were entered into the second block, and the interaction between intention and action planning was entered into the third block. Intention accounted for 3.1% (Adjusted *R*^2^ = 0.031, *p* = 0.003) of the variance in behavior and was a significant predictor of behavior (*β* = 0.19, *SE* = 14.59, *t* = 3.03 *p* = 0.003). The addition of action planning, implicit attitudes, and implicit motivation accounted for an additional 0.3% (Δ*R*^2^ change) of the variance in behavior above and beyond intention (Adjusted *R*^2^ = 0.034, *p* = 0.012). Only intention significantly predicted behavior (*β* = 0.16, *SE* = 15.17, *t* = 2.46, *p* = 0.015). Action planning (*β* = 0.11, *SE* = 32.56, *t* = 1.66, *p* = 0.097), implicit attitudes (*β* = 0.04, *SE* = 43.09, *t* = 0.61, *p* = 0.545), and implicit motivation (*β* = −0.07, *SE* = 62.81, *t* = −1.07, *p* = 0.285) did not significantly predict behavior. The addition of the interaction between intention and action planning in step three accounted for no additional variance in exercise behavior (Adjusted *R*^2^ = 0.034, *p* = 0.017). When the interaction term was included, none of the variables significantly predicted behavior: intention (*β* = −0.22, *SE* = 89.42, *t* = −0.57, *p* = 0.567), action planning (*β* = −0.04, *SE* = 81.98, *t* = −0.26, *p* = 0.793), implicit attitudes (*β* = 0.04, *SE* = 71.29, *t* = 0.54, *p* = 0.588), implicit motivation (*β* = −0.07, *SE* = 62.81, *t* = −1.08, *p* = 0.280), and the interaction between intention and action planning (*β* = 0.45, *SE* = 22.11, *t* = 1.01, *p* = 0.316). In the analysis utilizing the week 1 data with multivariate outliers included, intention was no longer significant at the second step. Otherwise, results were consistent across the entirety of the multiverse analysis in terms of significant predictors. Overall, these results highlight that the additional proximal predictors in the IBCM did not predict behavior better than intentions in the current data.

## Discussion

Behavioral theories have largely taken a social cognitive approach specifying intention as a proximal predictor of behavior, including only reflective constructs, thereby neglecting to include the influence of automatic processes, and have specified an invariant theory of behavior across different stages of behavior change such as initiation and maintenance. This is problematic as the intention-behavior gap is a well-observed phenomenon (e.g., Sheeran and Webb, 2016), focusing only on reflective factors, such as attitudes, ignores the influence that automatic processes have on behavior (Rebar et al., 2016), and predictors of behavior are known to differ between initiators and maintainers (Phillips et al., 2016). The overarching purpose of this study was twofold. First, the present research compared, two theories of health behavior change – for exercise initiators and maintainers – which are rooted in the link between intentions and behavior: the IBCM (Hagger and Chatzisarantis, 2014b) and its theoretical predecessor, the TPB (Fishbein and Ajzen, 2011). Second, this research compared the utility of the IBCM for predicting behavior against the TPB for initiators.

In this first known test of the IBCM for physical activity (Hagger and Chatzisarantis, 2014b), the utility of the IBCM was supported for people who were just beginning their exercise journey (i.e., initiators), and was more highly supported for initiators in comparison with maintainers. However, the IBCM did not add any unique contribution to the direct prediction of behavior in comparison with the TPB for initiators. That is, action planning, the interaction between action planning and intention, implicit attitudes, and implicit motivation did not predict behavior. This is in line with previous tests of the IBCM for other health behaviors (i.e., action planning: Hagger et al., 2017; Hamilton et al., 2017; Brown et al., 2018). Action planning has been shown to be a viable target to elicit behavior change in past intervention studies and is one of the only known techniques that leads to continued activity engagement six months post-intervention (Gollwitzer and Sheeran, 2006; Howlett et al., 2019). The lack of significant results concerning action planning in present and past tests of the IBCM may be due to the variability in the quality of plans (e.g., I will exercise on Fridays, versus I will exercise after work on Fridays; de Vet et al., 2011). The quality of plans may be especially problematic when made without the guidance provided by an intervention.

Neither implicit attitudes nor implicit motivation predicted physical activity. To that end, it is possible that implicit attitudes and motivation are not powerful targets for an intervention due to their null or small influence on behavior. This is likely to be especially true for implicit motivation, which is a trait-like tendency underpinning why one performs behaviors that is likely resistant to change. It should be noted that past research has intervened upon implicit attitudes with success in changing health behaviors (i.e., alcohol consumption, healthy eating practices) over a short period of time (Houben et al., 2010; Hollands et al., 2011). More research will be needed to test the viability of targeting implicit attitudes for sustained behavioral changes.

In contrast to the TPB, the IBCM adds autonomous motivation as a new distal target of intention formation. In the present study, this was supported through the mechanisms of perceived behavioral control and affective attitudes. However, greater theoretical development and empirical evaluation is needed regarding the causal relationships between motivation and other antecedents of behavior; namely, for physically taxing behaviors, like physical activity engagement, it is unlikely that initial engagement is ‘enjoyable’ or ‘fun’, especially for individuals who do not already have good cardiorespiratory fitness (Rhodes, 2017). Indeed, previous research has found that physical fatigue both during and after physical activity sessions is one of the most frequently reported barriers to engagement (Ebben and Brudzynski, 2008). Moreover, reasons for starting activity engagement are vast and extend beyond enjoyment—for example improving one’s physical appearance has been shown to be the most highly ranked reason across the lifespan (Gavin et al., 2014). Thus, the most autonomous form of motivation (i.e., intrinsic) in and of itself may not be a viable target in terms of changing intentions and subsequent behavior for most individuals as it likely does not reflect their pre-existing goals. Additionally, because feeling competent is a theoretical precursor of the development of fully autonomous or intrinsic motivation in Self-Determination Theory (Ryan and Deci, 2000), it is unlikely that fully autonomous motivation would precede perceived behavioral control as feeling competent requires behavioral practice. However, it should be noted that the causal link between autonomous motivation and attitudes, social norms, and perceived behavioral control as specified in the IBCM may be more theoretically appropriate when considering partially controlled motivations such as identified motivation (i.e., valuing the outcomes of a behavior).

Additionally, it is important to note that although the IBCM hypothesizes that action planning moderates the intention behavior gap, a recent systematic review has shown that many psychosocial variables are potentially important moderators of this relationship (Rhodes et al., 2022). These include demographic variables (e.g., employment), personality variables (e.g., conscientiousness), and automatic factors (e.g., identity). Moreover, another recent systematic review has shown that unpleasant experiences while engaging in physical activity may reduce participation for people with chronic illnesses that are related to increased pain and fatigue (Collado-Mateo et al., 2021). Thus, the TPB and IBCM likely need to be extended to include other moderators of the intention-behavior gap. These moderators may serve to not only identify who is at risk of not fulfilling their physical activity intentions (e.g., based on demographic factors, personality factors, and/or chronic illness status), but also propose mechanisms of maintenance – such as identity (Rhodes et al., 2016) – within traditional social cognitive frameworks.

The present study is not without limitations. First, the desired sample size was not collected due to the restrictions that were placed on data collection by the COVID-19 pandemic. Continuing data collection during the pandemic was deemed inappropriate given the contextual shifts that occurred, which had the capacity to undermine exercise behaviors. In support of this, 30% of individuals surveyed in the United States reported that they exercised less than usual during April of 2020 (Gough, 2020). Second, participants in the present study were college students who are unlikely to be representative of the general population (Peterson, 2001; Henrich et al., 2010). However, it is still important to understand the mechanisms of physical activity initiation and maintenance in students as approximately half of American college students are not sufficiently active (Keating et al., 2005). Third, the current study artificially dichotomized individuals based on their score on a stage of change measure and in accordance with the Transtheoretical Model, which may have resulted in range restriction (Prochaska and DiClemente, 1982; Sackett et al., 2007). In the current study, ‘maintainers’ engaged in more physical activity per week and had been engaging in physical activity for more months than ‘initiators’, which provides at least some evidence that these groups were different with regard to their behavior. Fourth, the current research, similar to past studies using the IBCM as a guiding framework, was observational. Therefore, causal inferences cannot be made from the results based on the present data as it can only be concluded whether variables were related. Finally, IATs were used to measure implicit attitudes and motivation. Although IATs are commonly used to measure implicit constructs (e.g., Project Implicit hosted by Harvard University), including in previous tests of the IBCM, they are also known to have several limitations. Specifically, the use of difference scores is psychometrically problematic as they are subject to higher type 1 error rates than non-difference scoring procedures (Edwards, 2001; Cafri et al., 2010). Additionally, previous research has highlighted that IAT scores tend to be weak predictors of behavior, which could be due to the measurement procedure itself or the scoring procedure as outlined above (Oswald et al., 2013). Moreover, traditional IATs cannot provide the refinement of measuring types of attitudes or motivation toward an activity beyond a mere dichotomy. Previous research using a single category IAT has assessed instrumental and affective attitudes toward activity behaviors (Phipps et al., 2021) and has found that implicit affective attitudes significantly predict physical activity, whereas implicit instrumental attitudes do not. Thus, it is possible that the IAT used in the present study was not sensitive enough and that future research using the IBCM should delineate the implicit constructs further. However, other research has provided evidence that IAT scores are more reliable than other implicit measures in terms of both test-retest and split-half reliability (Nosek et al., 2007; Znanewitz et al., 2018). Additionally, IAT scores also have been shown to have both convergent and divergent validity using multi-trait, multi-method matrices (Nosek and Smyth, 2007; Nosek et al., 2007).

There is a need for researchers to continue to refine and develop theories of behavior change to specify viable intervention targets for initiators, including automatic targets, and for including a maintenance phase of behavior change where appropriate targets are specified (Sheeran and Webb, 2016; Rothman, 2000). The IBCM is an attempt to improve the toolbox of targets for behavior change to include automatic processes, action planning, and autonomous motivation (Hagger and Chatzisarantis, 2014b). However, in the present study it was found that the IBCM did not improve upon its theoretical predecessor – the TPB (Fishbein and Ajzen, 2011). That is, the IBCM does not add any unique proximal predictors of behavior. Additional theories, such as the Health Action Process Approach, which also includes action planning, will need to be assessed as it already delineates initiation from maintenance (Schwarzer, 2016). However, dual-phase theories, including the Health Action Process Approach, will need to be extended to include automatic mechanisms of maintenance (e.g., habit) in addition to the reflective determinants that are already included. Moreover, research should assess whether automatic determinants of initiation add a unique contribution to behavior. However, it should be noted that in the present study automatic determinants of initiation did not contribute to the variance predicted in behavior.

The purpose of the present study was twofold. First, the present research compared, two theories of health behavior change – for exercise initiators and maintainers – which are rooted in the link between intentions and behavior: the IBCM (Hagger and Chatzisarantis, 2014b) and its theoretical predecessor, the TPB (Fishbein and Ajzen, 2011). Second, this research compared the utility of the IBCM for predicting behavior against the TPB for initiators. Although there are important limitations that need to be considered when interpreting the results, the present study highlights some important considerations for the field of behavior change. First, the mechanisms of behavioral engagement differed between initiators and maintainers. This is not the first study to support this delineation (e.g., Phillips et al., 2016), nor is it likely to be the last. Theories such as the TPB and IBCM need to specify these differences using a dual-phase approach to allow for more precise behavioral prediction, but also to include appropriate intervention targets depending on stage of change. Second, the present study suggests that the TPB should be preferred over the IBCM, because it is more parsimonious. Although the IBCM adds action planning, a known technique to promote behavioral engagement, this is not a unique contribution in and of itself given that action planning has been added to the TPB by others as a method of reducing the intention-behavior gap (e.g., Norman and Conner, 2005). However, since interventions based on the TPB and other social cognitive frameworks – with the exclusion of the more recently added action planning – have been shown to be sub-optimal in terms of behavior change (e.g., Kinmonth et al., 2008; Sniehotta, 2009), it is of the utmost importance that theory refinement and development continues.

## Data availability statement

The raw data supporting the conclusions of this article will be made available by the authors, without undue reservation.

## Ethics statement

The studies involving human participants were reviewed and approved by Iowa State University Office of Research Ethics. The participants provided their written informed consent to participate in this study.

## Author contributions

KM and LP contributed to the conceptualization, design of the study, and writing of the manuscript. KM organized the dataset and performed the analyses. Both authors contributed to manuscript revision and approved the current version.
